# Network Analysis of Depressive Symptoms and Meaning in Life and Their Association with Health-Related Quality of Life Among South Korean Older Adults

**DOI:** 10.3390/healthcare13182281

**Published:** 2025-09-12

**Authors:** Chanhee Kim, Heesung Ko

**Affiliations:** 1 Department of Nursing, Changwon National University, 20 Changwondaehak-ro, Uichang-gu, Changwon-si 51140, Gyeongsangnam-do, Republic of Korea; chany131@changwon.ac.kr; 2 Department of Nursing, Jesus University, 383 Seowon-ro, Wansan-gu, Jeonju-si 54989, Jeollabuk-do, Republic of Korea; 3 Department of Nursing, Daejeon University, 62 Daehak-ro, Dong-gu, Daejeon 34520, Republic of Korea

**Keywords:** depression, meaning in life, quality of life, network analysis, older adults

## Abstract

**Background/Objectives**: Depression is the most common mental health problem in older adults and is closely associated with meaning in life. This study aimed to investigate the interconnections between depressive symptoms and meaning in life and their association with health-related quality of life (HRQoL) in older adults using a network analysis. **Methods:** This cross-sectional study was performed between July and August 2021. The 10-item Center for Epidemiologic Studies Short Depression Scale (CES-D), the Meaning in Life Questionnaire, and the EQ-5D-3L were used. Central and bridge symptoms were identified using strength and bridge strength. Furthermore, a flow network was employed to examine whether depressive symptoms and meaning in life were associated with HRQoL. **Results**: Overall, 213 community-dwelling older adults participated. “Sadness,” “loneliness,” and “depressed” were found to be the central symptoms in the depressive symptoms and meaning in life network. “Presence of meaning” and “inability to get going” each had the highest bridge strength values in the depressive symptoms and meaning in life network. Moreover, “inability to get going”, “loneliness”, and “everything was an effort” had the strongest relationships with HRQoL. **Conclusions**: This study highlights the inter-relationships between depressive symptoms, meaning in life, and HRQoL. Interventions targeting the central and bridging symptoms are crucial for reducing depressive symptoms associated with a low sense of meaning in life among older adults. These findings offer insights to inform symptom-focused interventions aimed at reducing depressive symptoms (i.e., affective symptoms, loneliness, and motivational deficit) and at enhancing meaning in aging populations.

## 1. Introduction

Globally, the population of older adults is rapidly increasing, with South Korea projected to have over 40% of its population aged ≥65 years by 2050 [1]. Consequently, late-life depression has emerged as a critical societal challenge, exhibiting a prevalence rate of 13.5% among South Korean older adults [2]. This condition is associated with adverse outcomes, including physical comorbidities, functional impairment, and diminished quality of life [3,4]. However, late-life depression remains underdiagnosed and undertreated, highlighting the need for more effective approaches to address this concern [5].

Closely intertwined with depression in older adults is the construct of meaning in life, which is defined as the perception that one’s existence is significant and purposeful [6]. Meaning in life is an essential motivation for living [7] and plays a critical role in the well-being of older adults [8]. Meaning in life is conceptualized as comprising two dimensions: the presence of meaning—having a sense that life is meaningful—and the search for meaning—actively striving to find that meaning [6]. While the presence of meaning is consistently linked to less depressive symptoms in older adults [9,10,11], according to a recent meta-analysis, the search for meaning has shown mixed findings in relation to psychological distress depending on factors such as culture and age [12]. The distinct feature of presence and search for meaning on mental health remains underexplored, requiring further empirical investigation.

Health-related quality of life (HRQoL), encompassing physical, mental, and social well-being [13], is a critical indicator of successful aging [14]. Research has shown that depression and meaning in life are significantly related to HRQoL among older individuals [15,16,17]. Depression can affect HRQoL in several ways, including decreased functional capacity, increased physical morbidity, and social withdrawal [5,17,18]. Conversely, meaning in life may enhance HRQoL by promoting adaptive coping strategies, fostering social connectedness, and encouraging health-promoting behaviors [16].

Although these relationships have been well-established in the literature, the complex interplay between depressive symptoms, meaning in life, and HRQoL remains inadequately understood, particularly regarding the specific mechanisms through which these constructs influence one another.

Traditional approaches to depression conceptualize symptoms as interchangeable manifestations of an underlying latent variable, assuming that each depressive symptom equally reflects a common cause while overlooking symptom-specific effects and inter-relations [19,20,21]. Network theory in psychopathology offers a novel lens by conceptualizing mental disorders as systems of interacting symptoms rather than manifestations of an underlying latent variable [19,20,21]. This approach allows for the identification of central symptoms—those with the strongest connections to other symptoms—and bridge symptoms that link distinct symptom communities [22,23,24]. Unlike latent variable approaches, network analysis acknowledges symptom heterogeneity and highlights clinically relevant nodes for targeted intervention [21]. Through mapping symptom-level connectivity, this methodology explains how discrete depressive symptoms exert differential effects on distinct psychological domains, including meaning in life and QoL.

Despite increasing interest in network models of depressive symptoms, few studies have explored how specific depressive symptoms interact with meaning in life and how these relationships collectively influence HRQoL in older adults. Given the high prevalence of depressive symptoms with lack of meaning in life and the risk of diminished HRQoL among older adults, investigating the interplay between these variables using a network analysis would be beneficial. Such analysis can elucidate central and bridge symptoms that may serve as efficient intervention targets, thereby enhancing both mental health and HRQoL in aging populations. Therefore, this study aimed to investigate (1) the network structure of depressive symptoms and meaning in life (presence and search for meaning) and (2) examine whether these constructs relate to HRQoL among older adults. We hypothesized that distinct central and bridge symptoms would emerge, offering insights into intervention priorities for promoting late-life mental health.

## 2. Materials and Methods

### 2.1. Participants and Procedures

This study is a secondary analysis of data from a previously conducted research study [9] that examined the relationships between loneliness and depressive symptoms and the mediating effects of meaning in life among older adults. Data were collected during the coronavirus disease 2019 (COVID-19) pandemic from July 2021 to August 2021. Participants were community-dwelling older adults aged ≥65 years. The inclusion criteria for participation in this study were as follows: (1) individuals aged ≥65 years, (2) residing in community settings such as their own homes or other non-institutional environments, and (3) possessing sufficient cognitive and communicative ability to engage in the survey process and provide written informed consent independently. Participants were recruited through convenience sampling from several community senior centers located in the metropolitan cities of Busan and Daegu, South Korea. After explaining the purpose of the study, a questionnaire was administered to participants who voluntarily agreed to participate. Data were collected by trained researchers using pen and paper. In this study, 244 participants were surveyed; however, only 213 were analyzed, owing to incomplete responses. This study received approval from the Institutional Review Board of D. University (IRB No. 2-1040709-AB-N-01-202105-HR-031-04).

### 2.2. Measures

Depressive symptoms were assessed using the Korean version of the Boston Form of the CESD-10, which was originally developed by Kohout et al. [25]. This scale has been employed in large-scale epidemiological studies, including the Korean Longitudinal Study of Aging, which targets the older adult population in South Korea. Participants rated how frequently they experienced symptoms during the prior week using a 4-point Likert scale ranging from 0 (never/rarely) to 3 (all the time), with higher scores indicating more depressive symptoms. The Cronbach’s alpha for the CESD-10 was 0.80 in this study.

To assess meaning in life, the Korean version of the Meaning in Life Questionnaire (MLQ) was used, which was developed by Steger et al. [6] and translated and validated by Won et al. [26]. This measure consists of two domains: presence and search for meaning. The presence of meaning refers to individuals’ perception that life is meaningful, whereas the search for meaning refers to individuals’ motivation to find meaning. This scale uses a 7-point Likert scale ranging from 1 (“absolutely untrue”) to 7 (“absolutely true”), with higher scores indicating a greater level of meaning in life. In a study conducted by Won et al. [26], Cronbach’s alpha for both the presence and search for meaning were 0.88. In this study, Cronbach’s alpha for the presence and search for meaning were 0.89 and 0.77, respectively.

HRQoL was assessed using the EQ-5D-3L profile [27], which has been widely used to assess participants’ general health status in five dimensions (mobility, self-care, usual activities, pain/discomfort, and depression/anxiety) at three levels: 1 (“no problems”), 2 (“some or moderate problems”), and 3 (“extreme problems”). The Cronbach’s alpha for the EQ-5D-3L was 0.79 in this study.

### 2.3. Data Analysis

All analyses were conducted using R (version 4.4.1; R Core Team, Vienna, Austria, 2024). To estimate and visualize the network structure, we employed the qgraph package (version 1.9.8) [28], which is specifically designed to generate graphical representations of psychometric networks. This package allows for the intuitive visualization of symptom interconnections [28]. In the network analysis of depressive symptoms and meaning in life (search for and presence of meaning), nodes indicate individual symptoms or meaning constructs and edges represent the relationships between them. Green edges indicate positive associations, whereas red edges indicate negative associations; the thickness of the edge represents the strength of the association. The Least Absolute Shrinkage and Selection Operator combined with the extended Bayesian Information Criterion was used to estimate the network. The approach enhanced the clarity and interpretability of the network model by decreasing the total number of edges.

To examine the roles of specific nodes within and between constructs, we assessed the strength and bridge strength. The packages “bootnet” version 1.6 [29] and “networktools” version 1.6.0 [30] were used to estimate these indices. The strength indicates the most influential nodes (i.e., depressive symptoms or meaning in life constructs) within the overall network, while bridge strength highlights nodes that connect different communities (e.g., depressive symptoms vs. meaning in life), which may be critical targets for clinical interventions. Then, we employed the flow function in qgraph to examine whether depressive symptoms and meaning in life (presence and search for meaning) were directly or indirectly associated with HRQoL.

Network stability was estimated via the correlation stability (CS-coefficient) for strength and bridge strength, with values above 0.25 considered stable [29]. To assess edge accuracy, a bootstrapped 95% confidence interval (CI) was employed. A narrower CI indicates a more accurate network. Bootstrapped difference tests were used to estimate the stability of edge weights and node strengths.

## 3. Results

The study participants had a mean age of 75.07 years (standard deviation = 6.60; range = 65–92 years). Among the participants, 60.1% were women, and 60.1% had a spouse. Over 71.8% were religious, and 42.8% had a high school education level or higher. Table 1 shows the descriptive statistics of the study variables.

**Figure 1:**
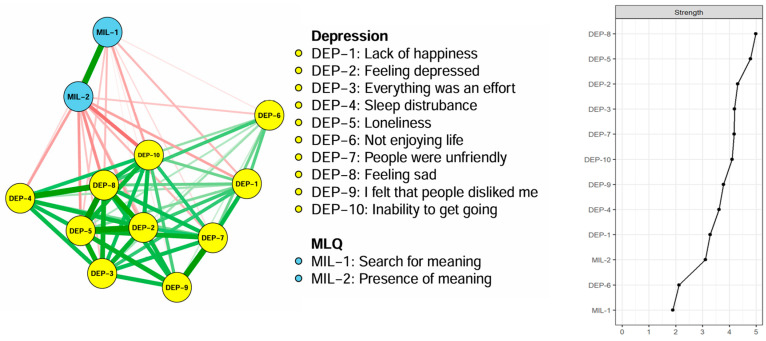
The network structure and centrality of depressive symptoms and meaning in life in older adults. MLQ: Meaning in Life Questionnaire; DEP-1: lack of happiness; DEP-2: feeling depressed; DEP-3: everything was an effort; DEP-4: sleep disturbance; DEP-5: loneliness; DEP-6: not enjoying life; DEP-7: people were unfriendly; DEP-8: feeling sad; DEP-9: I felt that people disliked me; DEP-10: inability to get going.

Figure 1 illustrates the network structure of depressive symptoms and meaning in life (presence and search for meaning). All items from the CESD-10 and meaning in life were entered into the network model because there were no redundant items.

The correlation matrix for the depressive symptoms and two dimensions of meaning in life is presented in Table S1. Most of the variables showed significant correlations; positive correlations ranged from 0.10 to 0.70, and negative correlations ranged from −0.44 to −0.10. Strong correlations between depressive symptoms and meaning in life (presence and search for meaning) were observed. The three strongest edges in depressive symptoms and meaning in life were “sadness”–“loneliness” (*r* = 0.69), “presence of meaning”–“search for meaning” (*r* = 0.69), and “unfriendly”–“disliked” (*r* = 0.69).

**Figure 2:**
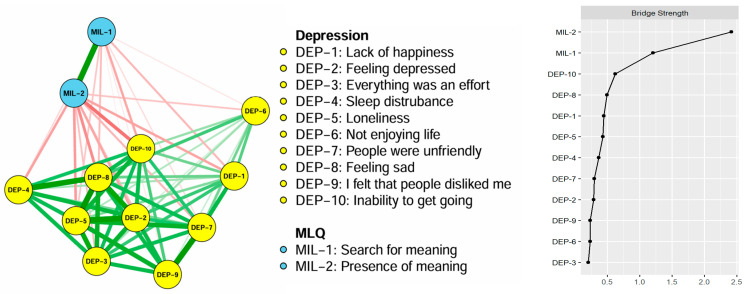
Bridge symptoms of depressive symptoms and meaning in life in older adults. MLQ: Meaning in Life Questionnaire.

On the right panel of Figure 1, the centrality strength ranking in the network model demonstrates that “sadness” had the highest strength value, followed by “loneliness” and “depressed,” indicating that these symptoms were the most influential in the depressive and meaning in life network model. Additionally, Figure 2 illustrates the bridge centrality for the network of depressive symptoms and meaning in life. In the depressive symptoms and meaning of life communities, “presence of meaning” and “inability to get going” each showed the highest bridge strength.

**Figure 3:**
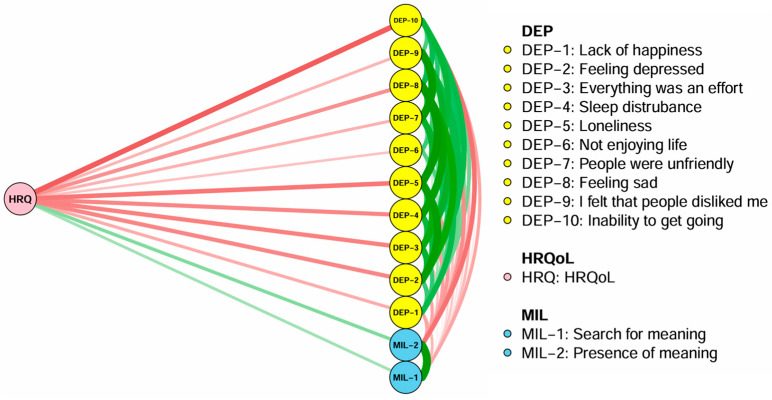
Flow network analysis of depressive symptoms and meaning in life with HRQoL in older adults. MLQ: Meaning in Life Questionnaire; HRQoL: health-related quality of life.

Figure 3 illustrates the flow network showing how depressive symptoms and meaning in life (presence and search for meaning) are connected to HRQoL within the network model. Twelve nodes remained in the flow network model. Among the nodes directly connected to HRQoL, “inability to get going,” “loneliness,” and “everything was an effort” had the strongest associations with HRQoL.

**Figure 4:**
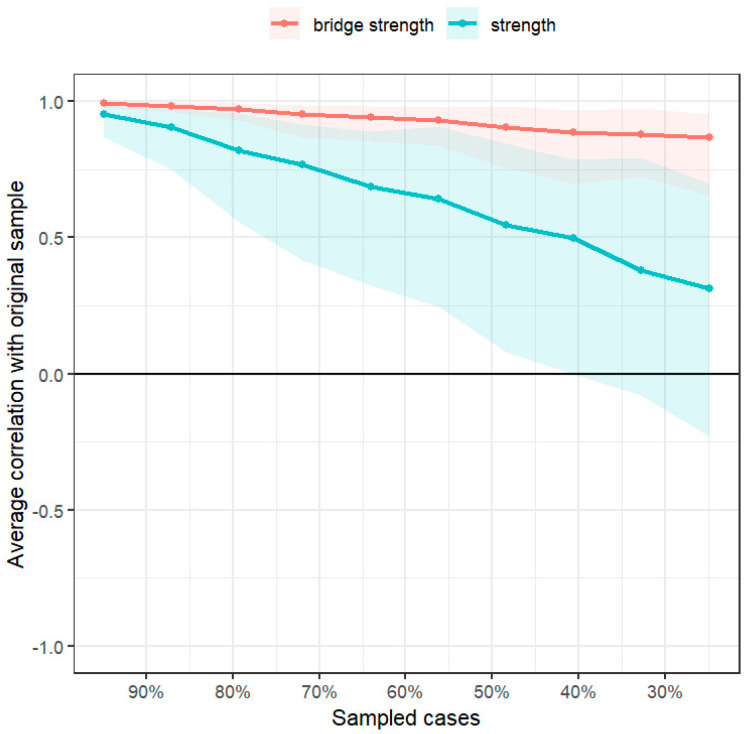
Stability tests of centrality and bridge centrality indices.

In the network stability analysis (Figure 4), the coefficients for centrality strength and bridge centrality strength, estimated using the case-dropping bootstrap method, were 0.129 and 0.673, respectively. The strength value was below the recommended minimum threshold of 0.25, indicating that the centrality estimates were unstable. The findings of the bootstrapped 95% CIs for the estimated edge and the bootstrapped difference test for edge weight and node strengths are presented in the Supplementary Material (Figures S1–S3).

## 4. Discussion

This study was conducted to explore a network model of depressive symptoms and meaning in life and their relationship with HRQoL among older South Korean adults during the COVID-19 pandemic.

In our study, the edge of “sadness–loneliness” showed the strongest association among all edges. This finding indicates that the association between affective symptoms and loneliness was stronger than associations among other depressive symptoms in our study population. This finding is consistent with that of previous network analyses of depressive symptoms that identified strong connections between sadness and loneliness [31,32]. This may be because loneliness is defined as perceived social isolation and usually encompasses painful negative emotions [33]; this emotional state is likely to lead to subsequent sadness or a depressive mood. Furthermore, the strong connection between loneliness and depressive symptoms is due to the fact that both feelings of loneliness and depressive mood symptoms commonly co-occur in older adults, reflecting the psychosocial context of aging, which is characterized by considerable life transitions such as diminished social networks, retirement, physical illness, and bereavement [33]. These age-related changes can simultaneously trigger feelings of sadness and loneliness, creating a mutually reinforcing relationship between these symptoms. Additionally, since this study collected data during the COVID-19 pandemic, the strict social restrictions may have contributed to a greater sense of loneliness and sadness in older adults. Therefore, helping lonely older adults may improve their sad mood, as targeting one symptom in each edge could simultaneously enhance their well-being [20].

Regarding central symptoms in the network of depressive symptoms and meaning in life, “sadness,” “loneliness,” and “depressed” had the highest centrality strength scores, indicating that these symptoms are the most influential compared with other depressive symptoms and meaning in life. Our findings are consistent with those of previous geriatric depression network studies. In a cross-sectional network analysis of depressive symptoms and life satisfaction among older South Korean adults conducted before the pandemic [34], “feeling depressed/blue” was identified as a central symptom. Similarly, “sad mood” was the core depressive symptom among older adults during the COVID-19 pandemic [35]. In another network analysis study by Bai et al. [36], “feeling depressed” was the central symptom in older Chinese adults. In addition, a longitudinal network analysis of depression in older adults showed that feeling depressed was the most consistently central symptom over time [37]. Similar findings suggest that the core affective symptoms (sadness and depressed mood) in older adults remain influential across time and different populations, even when factors such as meaning in life, life satisfaction, and cognitive function are included in the network analysis. Moreover, as one of the essential symptoms of major depressive disorders defined by the *Diagnostic and Statistical Manual of Mental Disorders* criteria, affective symptoms such as sad mood or depressed mood are considered high priority and can activate other depressive symptoms. Although geriatric depression is characterized by various atypical features [38], affective symptoms remain a central feature. In clinical practice, cognitive behavioral therapy targeting core affective symptoms such as sadness or feeling depressed may serve as a useful intervention for this population [39].

Another central symptom in the network of depressive symptoms and meaning in life was loneliness in our older population, indicating its important role in the entire network. In line with our findings, previous studies on depression networks have found that loneliness is one of the most central symptoms [40,41]. A systematic review examining the longitudinal relationship between loneliness and depressive symptoms revealed that loneliness was a predictor of subsequent changes in depressive symptoms [42]. In relation to the COVID-19 pandemic, loneliness has been a major concern among older adults [43] owing to preventive measures, such as lockdowns and quarantine, which might lead to depressive symptoms and a low sense of meaning in life. Given the strong centrality of loneliness in the network and its strong connections with affective symptoms, addressing loneliness is crucial for preventing depression and a diminished sense of meaning in life in older adults. Thus, reducing loneliness by enhancing social links among older adults may help diminish or prevent feelings of sadness and other depressive symptoms. Prior research has shown that a 4-week empathy-focused telephone program significantly improved loneliness, anxiety, and mental health in older adults during the pandemic [44]. Furthermore, a systematic review revealed that various technology-based interventions (companion robots, telephones, video conferences, and televisions with various applications) and community-engaged programs were effective in relieving loneliness among older adults [45]. Using these interventions to increase social links would be beneficial to address not only loneliness but also other depressive symptoms and lack of meaning in life among older adults.

Our study revealed that “inability to get going” and “presence of meaning” were identified as bridge symptoms, representing depressive symptoms and meaning in life. A recent network analysis conducted by Zhang et al. [46] revealed that in undergraduate students, sleep disturbances and anhedonia were key bridge symptoms across different levels of depression; this was not consistent with the findings of the present study. These discrepancies may be attributed to differences in the study populations—older adults versus university students—as well as variations in the data collection periods, specifically during the COVID-19 pandemic versus the post-pandemic phase. Similarly, a previous study identified “inability to get going” as a bridge symptom connecting depression to life satisfaction [34]. “Inability to get going” refers to a feeling of being unable to initiate or maintain activities with reduced motivation, often associated with low energy and fatigue [47]. The identification of “inability to get going” as a bridge symptom may reflect the profound impact of motivational deficits such as energy deficit or fatigue on an individual’s capacity to engage in meaningful activities and pursue purposeful goals, both of which are essential for maintaining a sense of meaning in life [6,7]. Notably, low energy and avolition may hinder the discovery of meaning in life through creative and experiential values, such as creating, working, and encountering people. Moreover, during the COVID-19 pandemic, older adults were forced to remain at home, and such strict restrictions may have reinforced avolition [48], leading to reduced meaningful social interactions and physical activity, ultimately resulting in a low sense of meaning in life. To address motivational deficits, integrating motivational interviewing with brief behavioral activation therapy could be an effective intervention strategy [49]. This combined approach may enhance older adults’ motivation to initiate and maintain engagement with behavioral activation, leading to improved clinical outcomes. Furthermore, the presence of meaning in life is another key bridge symptom associated with depression. Cumulative findings have revealed that having a sense of meaning in life is strongly associated with depressive symptoms [9,10,11,50,51]. Frankl [7] stated a perceived lack of meaning as an “existential vacuum,” which leads to feelings of emptiness and hopelessness—both central features of depression. The bridge role of the presence of meaning likely reflects its function as a psychological resource that buffers against negative impacts [8], such as age-related losses, challenges, and even pandemic crises. When older adults maintain a sense that their lives are meaningful despite difficulties, they may be less vulnerable to developing depressive symptoms in response to stressors. Additionally, the presence of meaning may facilitate adaptive cognitive appraisals of challenging situations [52], thereby reducing the likelihood that these experiences will trigger depressive symptoms. These findings highlight the potential value of interventions that explicitly focus on enhancing the sense of meaning in life, such as meaning-centered programs [53,54] or logotherapy-based approaches [55,56,57], which may effectively disrupt the pathways between existential concerns and depressive symptomatology.

Frankl [7] proposed three pathways to discovering meaning in life: creative, experiential, and attitudinal. For older adults, meaning in life may be fostered through engaging in meaningful activities (creative), nurturing relationships (experiential), and maintaining a resilient attitude in the face of adversity (attitudinal). In the context of South Korea, where community senior community centers are well established, active participation in educational programs, social links, and volunteering programs offered through these centers may serve as practical strategies for discovering and enhancing meaning in life. Such engagement aligns with Frankl’s three pathways by enabling older adults to contribute, connect with others, and cultivate a meaningful perspective in daily life. Therefore, providing targeted interventions such as motivational interviewing and logotherapy may not only address key bridging symptoms but also indirectly alleviate other interconnected depressive symptoms. In addition, identifying and monitoring these bridge symptoms in clinical practice can enhance assessment accuracy by revealing the pathways through which symptoms interact and exacerbate each other.

“Inability to get going,” “loneliness,” and “everything was an effort” were the top three symptoms related to HRQoL. Depressive symptoms such as “inability to get going” and “everything was an effort” reflect fatigue, diminished energy, and reduced motivation, which are common in older adults [5]. The finding that fatigue and a lack of motivation is significantly associated with poor HRQoL among older adults is supported by previous studies [14,58]. In a network analysis study involving adults with or at risk of knee osteoarthritis, physical HRQoL emerged as a bridge node linking depressive symptoms [59]. Similar to our study, in a network study of patients with Parkinson’s disease, “loss of energy” and “tiredness” or “fatigue” were found to bridge depressive symptoms and overall QoL [60]. These symptoms (fatigue, loss of energy, and avolition) can limit engagement in daily activities, reduce social participation, and exacerbate functional decline [61], all of which are associated domains measured by the EQ-5D instrument. Moreover, loneliness was closely associated with HRQoL among older adults, consistent with previous study findings [62]. This finding is attributed to the fact that loneliness undermines not only social functioning but also general health, physical functioning, mobility, and mental health [63,64], all of which may affect HRQoL domains. Therefore, our flow network analysis results suggest that interventions targeting motivational symptoms and loneliness may be effective in improving HRQoL in older adults. To address fatigue and diminished motivation, along with the previously mentioned motivational interviewing-based program, physical activity programs could be effective strategies in older adults. In a recent meta-analysis study [65], physical activity interventions for adults with chronic conditions were found to be effective in reducing fatigue. In terms of loneliness, programs aimed at improving social links (described above) may help older adults to address loneliness and enhance their HRQoL.

Some limitations should be considered when interpreting our findings. First, our study participants were community-dwelling older adults residing in urban areas of South Korea. Therefore, the findings may not be generalizable to institutionalized older adults or those living in rural regions. Furthermore, given the cultural context in which the study was conducted, caution is needed when applying these findings to older adults in other countries. Future studies involving larger and more diverse samples—across both residential settings and cultural backgrounds—are needed to confirm and extend these findings. Second, data collection was conducted during the COVID-19 pandemic, which limits the generalizability of our findings to the post-pandemic period. To examine whether our finding is valid in the post-pandemic era, further replication studies or a longitudinal study will be needed. Third, while the present study’s cross-sectional design limits causal inference, previous studies have shown a bidirectional relationship between depressive symptoms and meaning in life [66]. To elucidate these causal pathways, future longitudinal studies are needed. Specifically, employing methods such as the cross-lagged panel network model can capture the temporal and reciprocal influences between depressive symptoms and meaning in life over time. Fourth, although the sample size may limit generalizability, it falls within acceptable bounds for estimating psychological networks of this size [30], especially when regularization techniques are used. However, the relatively small sample size may have influenced the stability of the estimated network structure. Specifically, the CS-coefficient for centrality strength was below the recommended threshold, suggesting limited robustness of the centrality estimates. Although the network structure itself remains interpretable, caution is warranted when interpreting node centralities. Future studies with larger samples are required to enhance the generalizability and stability of the network results. Fifth, the CES-D-10 does not cover the full range of geriatric depressive symptoms, such as appetite changes, poor concentration, psychomotor agitation, and suicidal ideation. This limited symptom coverage may have led to the underestimation of clinically significant depressive symptoms, and thus central or bridging symptoms might have been overlooked. Further studies would benefit from employing more comprehensive depression assessment scales, such as the 20-item CESD scale or the Patient Health Questionnaire-9. Lastly, as the present study relied on self-administered measures, which may introduce recall and reporting biases, future research using multi-informant reports will be needed.

Despite the limitations, this study has several important strengths that contribute to the understanding of mental health in older adults. In this study, the identification of “sadness,” “loneliness,” and “depressed” as central symptoms suggests that these symptoms serve as influential nodes that may maintain and propagate depressive symptoms as well as lack of meaning in life among older adults. Targeting such central symptoms may therefore yield broader therapeutic effects across the depressive symptoms and lack of meaning in life among older adults, making them high-priority targets for both assessment and treatment. Moreover, considering that older adults are vulnerable to both existential distress and depression, the identification of bridge symptoms, such as “inability to get going” and “presence of meaning”, provides valuable insights for clinicians aiming to develop integrated therapeutic approaches that address both affective and existential dimensions of well-being. Third, since “inability to get going,” “loneliness,” and “everything was an effort” were found to be strongly associated with lower HRQoL, this information provides effective strategies for prioritizing intervention targets to enhance mental health as well as HRQoL among older adults.

## 5. Conclusions

This study revealed inter-relationships between meaning in life, depressive symptoms, and HRQoL in older adults. Interventions targeting central symptoms (affective symptoms and loneliness) and bridge symptoms (inability to get going and presence of meaning) may reduce depressive symptoms and enhance the sense of meaning in life in this population. Furthermore, enhancing fatigue symptoms and addressing loneliness in older adults may reduce the negative impact of depression and a low sense of meaning in life on HRQoL.

## Figures and Tables

**Table 1:** Descriptive statistics of the study variables.

Construct/Nodes	Mean	SD	Minimum	Maximum
DEP-1: Lack of happiness	0.54	0.89	0.00	3.00
DEP-2: Feeling depressed	0.63	0.85	0.00	3.00
DEP-3: Everything was an effort	1.00	0.97	0.00	3.00
DEP-4: Sleep disturbance	1.02	1.01	0.00	3.00
DEP-5: Loneliness	0.78	0.97	0.00	3.00
DEP-6: Not enjoying life	0.96	1.18	0.00	3.00
DEP-7: People were unfriendly	0.42	0.76	0.00	3.00
DEP-8: Feeling sad	0.60	0.81	0.00	3.00
DEP-9: I felt that people disliked me	0.36	0.72	0.00	3.00
DEP-10: Inability to get going	0.80	0.93	0.00	3.00
Total CESD	0.71	0.55	0.00	2.80
MIL-1: Search for meaning	5.03	1.23	1.60	7.00
MIL-2: Presence of meaning	4.94	1.10	1.40	7.00
Total MIL	4.99	1.07	1.50	7.00

Abbreviation: SD, standard deviation.

## Data Availability

The data presented in this study are available on request from the corresponding author.

## Supplementary Materials

The following supporting information can be downloaded at https://www.mdpi.com/article/10.3390/healthcare13182281/s1, Table S1: Correlation matrix of the depressive symptoms and meaning in life; Figure S1: Bootstrapped 95% CIs for estimated edge weights; Figure S2: Estimation of edge weight difference by bootstrapped difference test; Figure S3: Estimation of node strength difference by bootstrapped difference test.

## Abbreviations

The following abbreviations are used in this manuscript:

HRQoL	Health-related quality of life
CESD	Center for Epidemiologic Studies Short Depression Scale
MLQ	Meaning in Life Questionnaire
CI	Confidence interval
COVID-19	Coronavirus disease 2019

## References

[1] Statistics Korea 2024 Elderly Statistic. Seoul (Korea). http://kostat.go.kr/portal/korea/kor_nw/2/1/index.board?bmode=read&bSeq=&aSeq=363362&pageNo=1&rowNum=10&navCount=10&currPg=&sTarget=title&sTxt=.

[2] Ministry of Health and Welfare 2020 Survey of the Elderly. https://www.mohw.go.kr/board.es?mid=a10411010100&bid=0019&act=view&list_no=366496.

[3] Oh D.J., Han J.W., Kim T.H., Kwak K.P., Kim B.J., Kim S.G., Kim J.L., Moon S.W., Park J.H., Ryu S.H. (2024). Association of depression with the progression of multimorbidity in older adults: A population-based cohort study. Am. J. Geriatr. Psychiatry Open Sci. Educ. Pract..

[4] Tsaras K., Tsiantoula M., Papagiannis D., Papathanasiou I.V., Chatzi M., Kelesi M., Kaba E., Fradelos E.C. (2022). The effect of depressive and insomnia symptoms in quality of life among community-dwelling older adults. Int. J. Environ. Res. Public Health.

[5] Zenebe Y., Akele B., W/Selassie M., Necho M. (2021). Prevalence and determinants of depression among old age: A systematic review and meta-analysis. Ann. Gen. Psychiatry.

[6] Steger M.F., Frazier P., Oishi S., Kaler M. (2006). The Meaning in Life questionnaire assessed the presence of and search for meaning in life. J. Couns. Psychol..

[7] Frankl V.E. (1959). The spiritual dimension in existential analysis and logotherapy. J. Individ. Psychol..

[8] Irving J., Davis S., Collier A. (2017). Aging with purpose: Systematic search and review of literature pertaining to older adults and purpose. Int. J. Aging Hum. Dev..

[9] Kim C., Chun J. (2024). “Meaning in Life” mediates the relationship between loneliness and depressive symptoms among older adults during the COVID-19 pandemic. Healthcare.

[10] Dewitte L., Dezutter J. (2021). Meaning reflectivity in later life: The relationship between reflecting on meaning in life, presence and search for meaning, and depressive symptoms in older adults over the age of 75. Front. Psychol..

[11] Volkert J., Härter M., Dehoust M.C., Ausín B., Canuto A., Da Ronch C., Suling A., Grassi L., Munoz M., Santos-Olmo A.B. (2019). The role of meaning in life in community-dwelling older adults with depression and its relationship with other risk factors. Aging Ment. Health.

[12] He X.-X., Wang X.-Q., Steger M.F., Ji L.-J., Jing K., Liu M.-F., Ye B.-J. (2023). Meaning in life and psychological distress: A meta-analysis. J. Res. Pers..

[13] Revicki D.A., Kleinman L., Cella D. (2014). History of health-related QoL outcomes in psychiatry. Dialogues Clin. Neurosci..

[14] Eum M., Kim H. (2021). Relationship between active aging and quality of life in middle-aged and older Koreans: Analysis of the 2013–2018 KNHANES. Healthcare.

[15] Glaw X., Kable A., Hazelton M., Inder K. (2017). Meaning in life and meaning of life in mental health care: An integrative literature review. Issues Ment. Health Nurs..

[16] Hupkens S., Machielse A., Goumans M., Derkx P. (2018). Meaning in life of older persons: An integrative literature review. Nurs. Ethics.

[17] Mutai H., Obuchi K., Yokoi K., Furukawa T. (2024). Factors associated with health-related quality of life among community-dwelling older adults without social participation in Japan: A cross-sectional study. J. Appl. Gerontol..

[18] Sivertsen H., Bjørkløf G.H., Engedal K., Selbæk G., Helvik A.-S. (2015). Depression and quality of life in older adults: A review. Dement. Geriatr. Cogn. Disord..

[19] Borsboom D., Cramer A.O.J. (2013). Network analysis: An integrative approach to the structure of psychopathology. Annu. Rev. Clin. Psychol..

[20] Borsboom D. (2017). A network theory of mental disorders. World Psychiatry.

[21] Bringmann L.F., Albers C., Bockting C., Borsboom D., Ceulemans E., Cramer A., Epskamp S., Eronen M.I., Hamaker E., Kuppens P. (2022). Psychopathological networks: Theory, methods, and practice. Behav. Res. Ther..

[22] Jones P.J., Ma R., McNally R.J. (2021). Bridge centrality: A network approach to understanding comorbidities. Multivar. Behav. Res..

[23] Epskamp S., Borsboom D., Fried E.I. (2018). Estimating psychological networks and their accuracy: A tutorial paper. Behav. Res. Methods.

[24] Borsboom D. (2022). Reflections on an emerging new science of mental disorders. Behav. Res. Ther..

[25] Kohout F.J., Berkman L.F., Evans D.A., Cornoni-Huntley J. (1993). Two shorter forms of the CES-D (Center for Epidemiological Studies Depression) depression symptoms index. J. Aging Health.

[26] Won D., Kim K., Kwon S.-J. (2005). Validation of the Korean version of meaning in life questionnaire. Hanguk Simni Hakhoe Chi Kongang.

[27] Brooks R., Rabin R.E., de Charro F., Brooks R., Rabin R., de Charro F. (2003). The Measurement and Valuation of Health Status Using EQ-5D: A European Perspective.

[28] Epskamp S., Cramer A.O.J., Waldorp L.J., Schmittmann V.D., Borsboom D. (2012). qGraph: Network visualizations of relationships in psychometric data. J. Stat. Softw..

[29] Epskamp S., Fried E.I. (2018). Tutorial on regularized partial correlation networks. Psychol. Methods.

[30] Jones P. Networktools: Tools for Identifying Important Nodes in Networks [Computer Software]. https://CRAN.R-project.org/package=networktools.

[31] Mullarkey M.C., Marchetti I., Beevers C.G. (2019). Using network analysis to identify central symptoms of adolescent depression. J. Clin. Child Adolesc. Psychol..

[32] Yang M., Wei W., Ren L., Pu Z., Zhang Y., Li Y., Li X., Wu S. (2023). How loneliness is linked to anxiety and depression: A network analysis of Chinese university students. BMC Public Health.

[33] Mayerl H., Stolz E., Freidl W. (2023). Lonely and depression in older age: Prospective associations and common vulnerabilities. Aging Ment. Health.

[34] Jeong D., Shim E.-J. (2019). Network Analysis of the Association between Depressive Symptoms and Life Satisfaction among Older Adults. Korean J. Couns. Psychother..

[35] Jin Y., Sun H.L., Lam S.C., Su Z., Hall B.J., Cheung T., Qin M.Z., Ng C.H., Xiang Y.T. (2022). International Research Collaboration on COVID-19. Depressive symptoms and sex differences among older adults in Hong Kong during the COVID-19 pandemic: A network analysis approach. Int. J. Biol. Sci..

[36] Bai W., Zhang J., Smith R.D., Cheung T., Su Z., Ng C.H., Zhang Q., Xiang Y.-T. (2023). Inter-relationship between cognitive performance and depressive symptoms and their association with quality of life in older adults: A network analysis based on the 2017–2018 wave of Chinese Longitudinal Healthy Longevity Survey (CLHLS). J. Affect. Disord..

[37] Sun H.-L., Zhang Q., Si T.L., Bai W., Chen P., Lam M.I., Lok K.-I., Su Z., Cheung T., Ungvari G.S. (2024). Interactive changes in depression and loneliness symptoms before and during the COVID-19 pandemic: A longitudinal network analysis. Psychiatry Res..

[38] Aziz R., Steffens D.C. (2013). What are the causes of depression later in life?. Psychiatr. Clin. N. Am..

[39] Wrede N., Hillebrand M.C., Risch A.K., Alpers G.W., Bartholdy S., Brakemeier E.L., Bräscher A.K., Brockmeyer T., Christiansen H., Equit M. (2025). How do young-old and old-old adults benefit from cognitive-behavioral therapy compared to working-age adults? A large multicenter naturalistic study. J. Affect. Disord..

[40] Gijzen M.W.M., Rasing S.P.A., Creemers D.H.M., Smit F., Engels R.C.M.E., De Beurs D. (2021). Suicidal ideation as a symptom of adolescent depression. A network analysis. J. Affect. Disord..

[41] Schlechter P., Ford T.J., Neufeld S.A.S. (2023). Development of depressive symptoms in older adults from a network perspective in an English longitudinal study of aging. Transl. Psychiatry.

[42] Van As B.A.L., Imbimbo E., Franceschi A., Menesini E., Nocentini A. (2022). Longitudinal association between loneliness and depressive symptoms in the elderly: A systematic review. Int. Psychogeriatr..

[43] Dahlberg L., Hajek A., Riedel-Heller S.G., König H.-H. (2024). Loneliness during the COVID-19 pandemic. Loneliness and Social Isolation in Old Age.

[44] Kahlon M.K., Aksan N., Aubrey R., Clark N., Cowley-Morillo M., Jacobs E.A., Mundhenk R., Sebastian K.R., Tomlinson S. (2021). Effect of layperson-delivered, empathy-focused program of telephone calls on loneliness, depression, and anxiety among adults during the COVID-19 pandemic: A randomized clinical trial. JAMA Psychiatry.

[45] Poscia A., Stojanovic J., La Milia D.I., Duplaga M., Grysztar M., Moscato U., Onder G., Collamati A., Ricciardi W., Magnavita N. (2018). Interventions targeting loneliness and social isolation among the older people: An update systematic review. Exp. Gerontol..

[46] Zhang S.S., Zhang W.H., Yong S.H., Chen J.T. (2025). Network analysis of meaning in life, perceived social support, and depressive symptoms among vocational undergraduate students. Front. Psychiatry.

[47] Kraft B., Bø R., Jonassen R., Heeren A., Ulset V.S., Stiles T.C., Landrø N.I. (2023). The association between depression symptoms and reduced executive functioning is primarily linked by fatigue. Psychiatry Res. Commun..

[48] Lebrasseur A., Fortin-Bédard N., Lettre J., Raymond E., Bussières E.-L., Lapierre N., Faieta J., Vincent C., Duchesne L., Ouellet M.-C. (2021). Impact of the COVID-19 pandemic on older adults: A rapid review. JMIR Aging.

[49] Balán I.C., Lejuez C.W., Hoffer M., Blanco C. (2016). Integrating motivational interviewing and brief behavioral activation therapy: Theoretical and practical considerations. Cogn. Behav. Pract..

[50] Li J.-B., Dou K., Liang Y. (2021). Relationship between the presence of meaning, the search for meaning, and subjective well-being: A three-level meta-analysis based on the Meaning in Life Questionnaire. J. Happiness Stud..

[51] Aftab A., Lee E.E., Klaus F., Daly R., Wu T.-C., Tu X., Huege S., Jeste D.V. (2019). Meaning in life and its relationship with physical, mental, and cognitive functioning: A study of 1,042 community-dwelling adults across their lifespan. J. Clin. Psychiatry.

[52] Park C.L. (2010). Making sense of the meaning literature: An integrative review of meaning making and its effects on adjustment to stressful life events. Psychol. Bull..

[53] Kang K.-A., Han S.-J., Lim Y.-S., Kim S.-J. (2019). Meaning-centered interventions for patients with advanced or terminal cancer: A meta-analysis. Cancer Nurs..

[54] Shen B., Liu J., Zhou Y., Zhu H. (2025). Effectiveness of meaning-centered interventions on anxiety and depressive symptoms, sense of meaning, and quality of life in patients with advanced cancer: A meta-analysis of randomized controlled trials. Support. Care Cancer.

[55] Dilmaghani R., Panahali A., Aghdasi A.N., Khademi A., Hajek A., Riedel-Heller S.G., König H.-H. (2024). Effectiveness of group logo therapy on death anxiety, feelings of loneliness, and meaning of life in elderly women with fear of coronavirus. Loneliness and Social Isolation in Old Age.

[56] Humer E., Kisler I.M., Bach D., Schimböck W., Pieh C., Probst T. (2025). Effect of a group logotherapeutic program for older adults on mental health indicators. J. Humanist. Couns..

[57] Kim C., Choi H. (2020). Efficacy of group logotherapy in community-dwelling older adults with depressive symptoms: A mixed-methods study. Perspect. Psychiatr. Care.

[58] Li X., Wang P., Jiang Y., Yang Y., Wang F., Yan F., Li M., Peng W., Wang Y. (2024). Physical activity and health-related quality of life in older adults: Depression as a mediator. BMC Geriatr..

[59] Solmi M., Koyanagi A., Thompson T., Fornaro M., Correll C.U., Veronese N. (2020). Network analysis of the relationship between depressive symptoms, demographics, nutrition, quality of life and medical condition factors in the Osteoarthritis Initiative database cohort of elderly North-American adults with or at risk for osteoarthritis. Epidemiol. Psychiatr. Sci..

[60] Heimrich K.G., Mendorf S., Schönenberg A., Santos-García D., Mir P., Group C.S., Prell T. (2023). Depressive symptoms and their impact on quality of life in Parkinson’s disease: An exploratory network analysis approach. J. Clin. Med..

[61] Soysal P., Veronese N., Thompson T., Kahl K.G., Fernandes B.S., Prina A.M., Solmi M., Schofield P., Koyanagi A., Tseng P.-T. (2017). Relationship between depression and frailty in older adults: A systematic review and meta-analysis. Ageing Res. Rev..

[62] Tan S.S., Fierloos I.N., Zhang X., Koppelaar E., Alhambra-Borras T., Rentoumis T., Williams G., Rukavina T., van Staveren R., Garces-Ferrer J. (2020). Association between loneliness and health-related quality of life (HR-QoL) among community-dwelling older adults. Int. J. Environ. Res. Public Health.

[63] Courtin E., Knapp M. (2017). Social isolation, loneliness, and health in old age: A scoping review. Health Soc. Care Community.

[64] Perissinotto C.M., Stijacic Cenzer I.S., Covinsky K.E. (2012). Loneliness in older persons: A predictor of functional decline and death. Arch. Intern. Med..

[65] Barakou I., Sakalidis K.E., Abonie U.S., Finch T., Hackett K.L., Hettinga F.J. (2023). Effectiveness of physical activity interventions on reducing perceived fatigue among adults with chronic conditions: A systematic review and meta-analysis of randomised controlled trials. Sci. Rep..

[66] Tam K.W., Zhang D., Li Y., Xu Z., Li Q., Zhao Y., Niu L., Wong S.Y. (2025). Meaning in life: Bidirectional relationship with depression, anxiety, and loneliness in a longitudinal cohort of older primary care patients with multimorbidity. BMC Geriatr..

