# Supplementary material for: Network Analysis of Depressive Symptoms and Meaning in Life and Their Association with Health-Related Quality of Life Among South Korean Older Adults
Source: Healthcare (Basel). 2025 Sep 12;13(18):2281. doi: 10.3390/healthcare13182281 (PMC12469548; doi:10.3390/healthcare13182281)
Supplement: Supplementary file 1 [file healthcare-13-02281-s001.zip › healthcare-3675639-SI.pdf]

Supplementary Table S1. Correlation matrix of the depressive symptoms and meaning in life

	HRQoL	MIL-1	MIL-2	DEP-1	DEP-2	DEP-3	DEP-4	DEP-5	DEP-6	DEP-7	DEP-8	DEP-9	DEP-10
HRQoL	1.000												
MIL-1	0.202	1.000											
MIL-2	0.254	0.686	1.000										
DEP-1	-0.231	-0.198	-0.248	1.000									
DEP-2	-0.331	-0.044	-0.244	0.359	1.000								
DEP-3	-0.343	-0.034	-0.169	0.362	0.518	1.000							
DEP-4	-0.328	-0.108	-0.260	0.196	0.486	0.461	1.000						
EP-5	-0.371	-0.158	-0.275	0.258	0.662	0.573	0.443	1.000					
DEP-6	-0.171	-0.072	-0.162	0.323	0.102	0.203	0.114	0.170	1.000				
DEP-7	-0.214	-0.099	-0.200	0.468	0.381	0.440	0.276	0.530	0.285	1.000			
DEP-8	-0.300	-0.183	-0.311	0.302	0.595	0.541	0.654	0.690	0.355	0.443	1.000		
DEP-9	-0.206	-0.065	-0.170	0.275	0.464	0.467	0.208	0.528	0.121	0.685	0.461	1.000	
DEP-10	-0.438	-0.242	-0.380	0.292	0.461	0.424	0.406	0.502	0.223	0.378	0.455	0.341	1.000

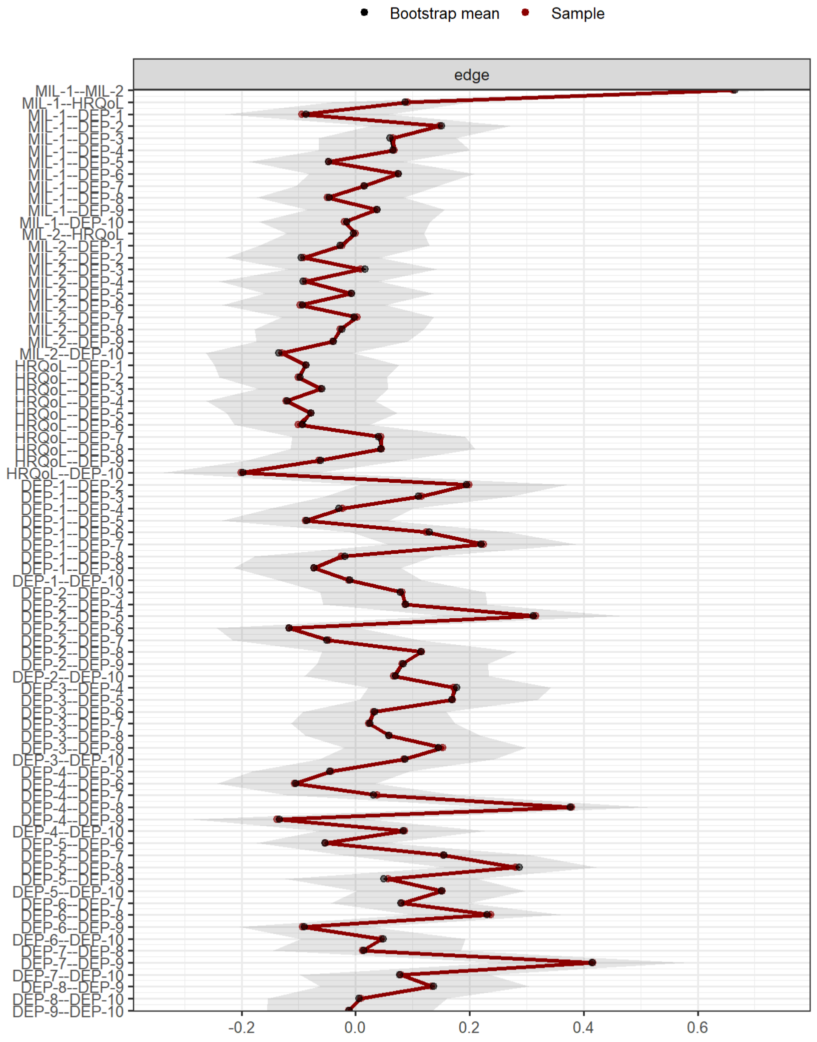

Supplementary Figure S1. Bootstrapped 95% CIs for estimated edge weights

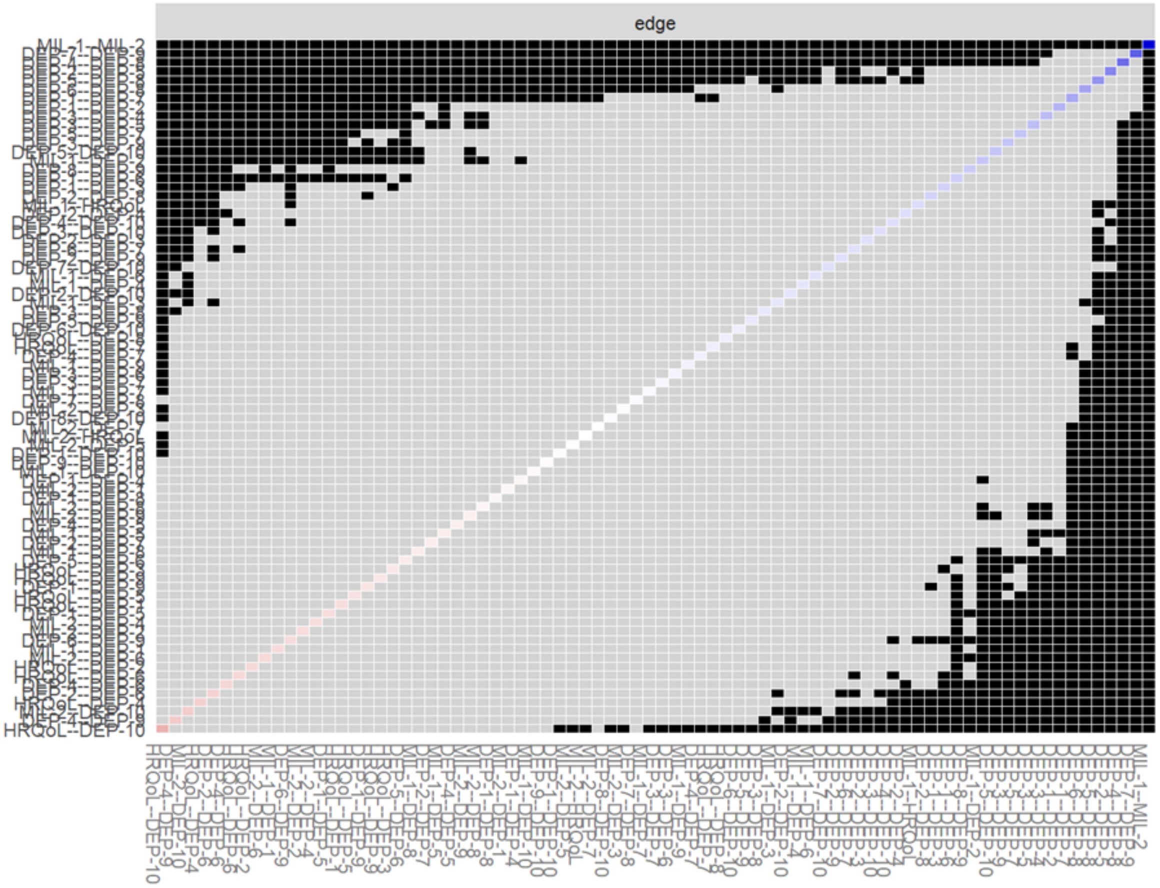

Supplementary Figure S2. Estimation of edge weight difference by bootstrapped difference test

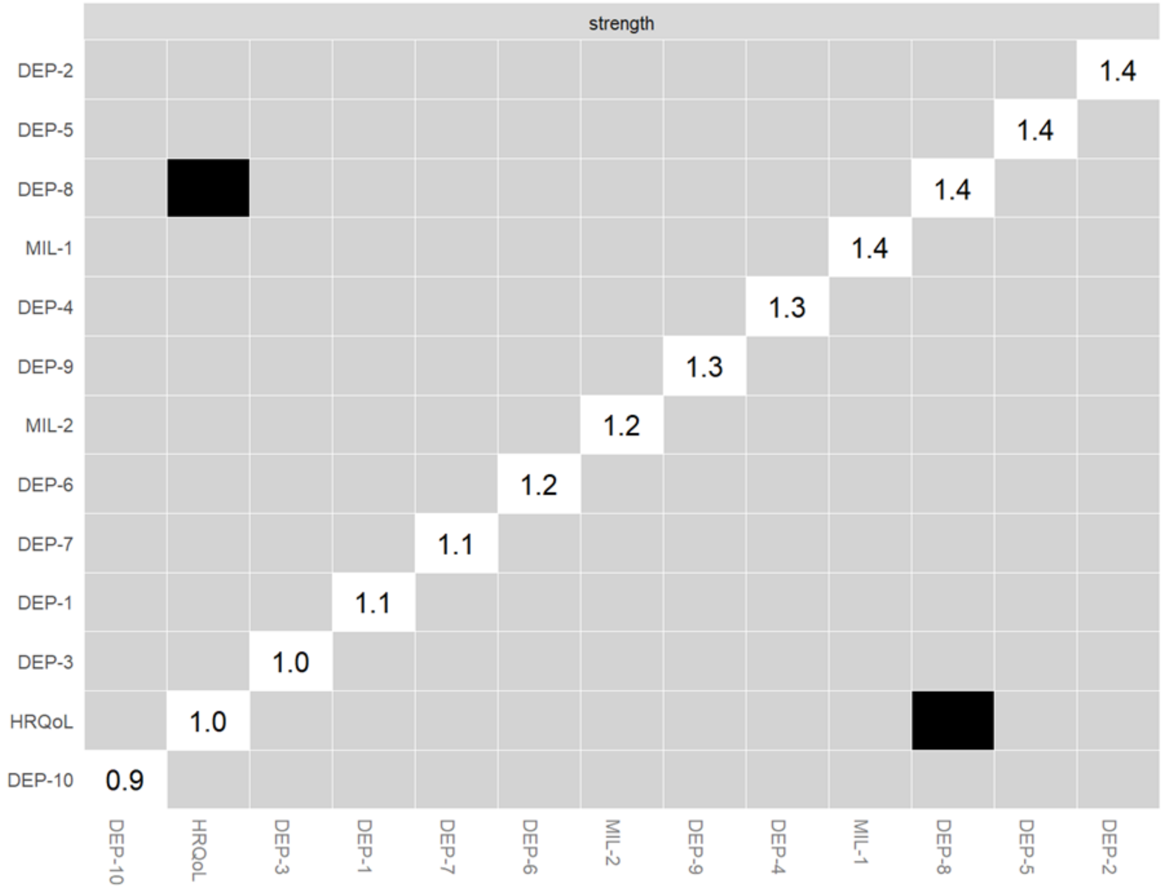

Supplementary Figure S3. Estimation of node strength difference by bootstrapped difference test
